# For Every Thing There is a Season and a Time …: The Construction of a Humanoid. A Tribute to Vincenzo Tagliasco

**DOI:** 10.3389/fbioe.2014.00019

**Published:** 2014-06-10

**Authors:** Danilo Emilio De Rossi, Annamaria D’Ursi

**Affiliations:** ^1^Research Center “E. Piaggio”, University of Pisa, Pisa, Italy; ^2^Aitek SpA, Genova, Italy

**Keywords:** humanoid, automa, machine, artifacts, builder

## Abstract

“There’s a time to be born, and a time to die; a time to break down, and a time to build up; a time to weep, and a time to laugh; a time to keep silence, and a time to speak…” (Ecclesiastes 3, 2–7). There was a time when automata were designed like clocks. Androids will have the time of their creators, the state of the art in technology, a wealth of experience to draw from, as well as the capacity to carry out actions as being endowed with meaning. The machine will undergo a long period of nurturing, from which it will learn to shape some sort of identity.

## Machines Will Lead Us to a New Kind of Humanism

### To Vincenzo Tagliasco (1941–2008), Pioneer in Humanoid Robotics and Artificial Counsciousness Studies

Rosenblueth and Wiener (1945), in *The Philosophy of Science* argued: “The best material model for a cat is another, or preferably the same cat.”

Stanislaw Lem (1961) in *Solaris*, his science fiction novel, writes: “We take off into the cosmos, ready for anything: for solitude, for hardship, exhaustion, death. We do not want to conquer the cosmos, we simply want to extend the boundaries of Earth to the frontiers of the cosmos. (…) We have no need of other worlds. We need mirrors. We do not know what to do with other worlds.”

Summing up the two quotations, which involve a scientific and science fiction approach, both Wiener’s “model” and Lem’s “mirror” refer to the concepts of replica, mimesis, reconciliation, and reduction of the same object or subject (Tagliasco, 1999).

The whole history of automata, which for the sake of convenience we symbolically trace back to Heron of Alexandria with his doves, had as its objective the attempt to reproduce and imitate. The ancient Greeks conceived of the mythical ambivalent Promethean will. On the one hand, this will push man to want to imitate the creative capacity of the gods, as a pure act of will, but also to exercise the power of a creator who retains control over his creatures. On the other hand, it entails the ability to steal the tools of knowledge from the gods, that is, to know how to create knowledge in order to facilitate survival, in building artifacts, including human simulacra, in order to empower their tools to perform difficult tasks, as if they were mechanical slaves.

According to Rinaldi (1981) in his introduction to the book of J. Cohen “in the Greek world the machine had a magical character since it opposes nature, it operates against nature and thus, given that the mechanician, the engineer, the mekanopoiòs is a creator of machines, he is, as a consequence, a wizard, a demon.”

In the Hellenistic period, an engineer is an ambiguous character, difficult to define; he does not care about theory, although he understands geometry and mathematics; he neither operates as an artisan nor as a technician. He is unable to place himself between *teknè* and *episteme* since the radicality of the contradiction makes mediation impossible. He becomes a pivot in the struggle with nature, which he forces, through his knowledge, to produce wonders.

The rationale behind new technologies at the beginning of the modern era does not lie in conceiving, inventing, and constructing machines because of their utility or economic value: this only happens later with the onset of the industrial revolution (Somenzi and Cordeschi, 1986). The aim instead, lies in the realization that nature could actually be controlled and dominated, that all possible machines could be built, that is, all machines that can be conceived in line with the new physics, to ascertain the possibility of making something from every other thing. This challenge reflects what Bergson (1907) says in his analysis of the natural function of intelligence related to instinct: “Qu’il nous suffise de dire que *l’intelligence est caractérisée par la puissance indéfinie de decomposer selon n’importe quelle loi et de recomposer en n’importe quel sistème*.”

The industrial society was a mass society with huge industrial enterprises and gigantic civil services, standardized mass production methods, and widespread conformism in the media and life styles. In this society, utilities/economicity was the engineering factor. Present information society is and will increasingly be a society of individuals in which new technologies will cope with the younger generation’s tendency to escape from a life of conformism and to express individuality. All this is occurring through the emergence of a social and economic order based on decentralized power and on the responsibility of the individual. On the other hand, the role of the playful/magic engineering factor will drive the construction of artifacts and machines (Florman, 1996).

So the early development of a new technology very much depends on the values recognized by leading social components of the time. The self-perception of their own role as operators involved in a technical invention also has an influence on the selection of the style and subject of the study (White, 1987).

It is a fact that in old times, in the construction of automata there was a magical intent, an alchemical influence, the desire for creationist power. A clear view of science and the experimental thrust of incremental technology afterward conditioned that story. The endless production of objects of robotics descends from the Promethean perspective: the act of creation, or more secularly the act of creating, exhausts the action.

The story unfolds into the wonders of androids, such as Geminoid F (Nishio et al., 2007), which recites Haikus on loneliness, designed to “articulate feelings” given that it is conceived as being a robot companion for the elderly. Laboratory practice is also not lacking in the Promethean tradition of constructing automata, i.e., construction as hypothesis testing, but also the pleasure of being able to amaze the public by exhibiting machines that know how to do things on their own.

Question of why a humanoid should be built, and furthermore what drives the human beings to build a humanoid, leads to question about how humanoids have to be built.

A careful analysis of the state of art suggests some directions that the methods of construction will take (Lipson and Pollack, 2000; Pfeifer et al., 2007).

It is very interesting that scientific progress in all those disciplines that converge in robotics does not only pose a technological paradigm shift, as discussed by Brooks (2002).

The construction of a humanoid, in some way, forces us to revise the mythological origins of the underlying reasons that drive a researcher to take part in a project. The development of science and technology enabling the construction of humanoids, has also helped to change the working practices of the builders of humanoids in the course of epigenetic (Lungarella et al., 2003) or developmental, individual growth (Floreano and Nolfi, 2000).

Genetic and evolutionary algorithms (Goldberg, 1989) have provided tools to drive robot evolutionary, competitive development (Nolfi et al., 1994).

However, if we attempt to adopt a hard biomimesis approach, we would proceed along the lines set out by Parisi (1993).

A true characteristic of living systems resides in the emerging nature of their behavior, which in itself prevents any prior deterministic reasoning on the structures and function of the entity to be generated.

In fact, as Parisi says: “A form of engineering truly inspired by biology should aim to construct artifacts which, when left in the hands of the engineer, are not yet what they should be, but will become so after a process of development. Engineers no longer create the artifact, they just create the conditions for an artifact to develop and learn.”

What about these conditions? In the Promethean paradigm, automata were designed like clocks. They repeated always the same performance, even if they were and are very complex mechanisms. Time was an external dimension.

As for human being, for a humanoid robot time should become an internal dimension. Which conditions are necessary for a humanoid to develop along a temporal line? How could a humanoid realize a personal story, an individual capacity to define a narrative line of its experiences? It is not just a question of software engineering to be able to consider and analyze the time in which the artifact lives. Like parents who try to understand how to educate and teach to their offspring what it is essential in their time, to interpret their personal experiences, social conditions, and visions of the world. Engineers have to conceive means to endow their creatures with the capacity to perform meaningful and responsible actions (Marino and Tamburrini, 2006).

What about meaning? Meaning is what a human being is able to recognize if an action is coherent with what others recognize as pertinent and appropriate, within historical, social, scientific, anthropological, mediatic contests. This is within relationships. It could be seen as a tautology, but to implement a series of codes leading to meaningful humanoid actions, unfolding in time, is the greatest challenge for an engineer.

In fact, staying in the realm of the myth of Prometheus who creates human beings, molding them from mud and animating them with divine fire, engineers create the artifact and mold the statue. But their task does not end there. The engineer is first a builder, then a “breeder of machines.” The focus shifts from an artificial being that can demonstrate syllogistic reasoning, to the capacity of this machine to learn and adapt. The creation of an artifact capable of learning and changing behavior over time affects the practice of an engineer in at least three ways: the personalization of artifacts, their “breeding,” and their uniqueness.

Engineers, by being “breeders of machines,” extend the relationship with their artifacts indefinitely, subjecting them to training, by measuring the capacity of machines to learn and develop autonomous behaviors, along a journey in which the ability of the designer to nurture determines the possibility of “growth” of the artifact.

An artifact that can learn and produce non-predefined behavior, as an outcome of processes that fit together in non-predetermined patterns dependent on the relationship with the outside world, is thus a unique and unrepeatable artifact, much closer to the artifacts of traditional craftsmanship or the work of an artist.

The power of the Prometheus myth fades to make way for another myth that seems better suited to explain this transition: the myth of Pygmalion. The act of creation or construction of the statue, whose beauty Pygmalion falls in love with, is not the completion of the action. The metamorphosis of the statue, which comes to life beginning to exhibit its nature, is not the result of the act of creation, but the effort that Pygmalion implements in shaping the characteristics that also make the statue a creative being. The artifact is subjected to a process of care, attention, and education, that becomes learned behavior; the metamorphosis produces a creature, an individual. The attitude of the builder comes close to a craftsperson who creates an object that will be unique and absolutely irreproducible, as in the production of a work of art that expresses its independence and uniqueness (Hofstadter, 2007).

Kelly (1994) says: “Yet as we unleash living forces into our created machines, we lose control of them. They acquire wildness and some of the surprises that the wild entails. This, then, is the dilemma all gods must accept: that they can no longer be completely sovereign over their finest creations. The world of the made will soon be like the world of the born: autonomous, adaptable, and creative but, consequently, out of our control.”

The fear of the loss of control also has ancient mythological roots. It is very well-represented in the legend of the Golem and then taken over by science fiction writers such as Isaac Asimov or by the playwright Karel Capek, or more recently by Dick (1996) with the rebel replicants of Blade Runner.

Regarding Kelly’s warning to all the gods that they can no longer have total sovereignty over their creatures, which can escape from our control due to their adaptability and creativity, the construction, and the breeding of a humanoid puts the engineer in a new light. From the dialectics of the two categories of power and control, which have dominated and driven technology, starting from the industrial revolution to the theory of servomechanisms of Wiener (1948) from which recent history unravels, an engineer’s focus shifts to the ability of the machine to evolve. A process of waiting and wondering what the machine will be able to express and what suggestions or hypotheses the machine itself will be able to arouse in the observer, be it the technologist, scientist, or the user of the machine itself. The machine’s performance will not be assessed in terms of the purpose for which it was designed, but on its level of autonomy. We could question as Goertzel (2013), in commenting Kurzweil (2006) forecasts: “Will machines take control?” We do not endorse this view. Machines will undergo a long and true processes of “care” from which they will learn to define some sort of identity, responsibility, and ethics, with a symbiotic liaison with the mentor, forced to operate accordingly on himself.

We do believe machines will lead us to a new kind of humanism.

## Conflict of Interest Statement

The authors declare that the research was conducted in the absence of any commercial or financial relationships that could be construed as a potential conflict of interest.
